# SNP in *PTPN22*, *PADI4,* and *STAT4* but Not *TRAF1* and *CD40* Increase the Risk of Rheumatoid Arthritis in Polish Population

**DOI:** 10.3390/ijms24087586

**Published:** 2023-04-20

**Authors:** Tomasz Budlewski, Joanna Sarnik, Grzegorz Galita, Grzegorz Dragan, Olga Brzezińska, Marta Popławska, Tomasz Popławski, Joanna Makowska

**Affiliations:** 1 Department of Rheumatology, Medical University of Lodz, 92-115 Lodz, Poland; 2 Department of Clinical Chemistry and Biochemistry, Medical University of Lodz, 92-215 Lodz, Poland; 3 Doctoral Study in Molecular Genetics, Cytogenetics and Medical Biophysics, Faculty of Biology and Environmental Protection, University of Lodz, 90-236 Lodz, Poland; 4 Biobank, Department of Immunology and Allergy, Medical University of Lodz, 92-213 Lodz, Poland; 5 Department of Pharmaceutical Microbiology and Biochemistry, Medical University of Lodz, 92-215 Lodz, Poland

**Keywords:** rheumatoid arthritis, *PADI4*, *STAT4*, *PTPN22*, single nucleotide polymorphisms, *rs2240340*, *rs7574865*, *rs2476601*

## Abstract

Single nucleotide polymorphisms in non-*HLA* genes are involved in the development of rheumatoid arthritis (RA). SNPS in genes: *PADI4* (*rs2240340*), *STAT4* (*rs7574865*), *CD40* (*rs4810485*), *PTPN22* (*rs2476601*), and *TRAF1* (*rs3761847*) have been described as risk factors for the development of autoimmune diseases, including RA. This study aimed to assess the prevalence of polymorphisms of these genes in the Polish population of patients with rheumatoid arthritis as compared to healthy controls. 324 subjects were included in the study: 153 healthy subjects and 181 patients from the Department of Rheumatology, Medical University of Lodz who fulfilled the criteria of rheumatoid arthritis diagnosis. Genotypes were determined by Taqman SNP Genotyping Assay. *rs2476601* (G/A, OR = 2.16, CI = 1.27–3.66; A/A, OR = 10.35, CI = 1.27–84.21), *rs2240340* (C/T, OR = 4.35, CI = 2.55–7.42; T/T, OR = 2.80, CI = 1.43–4.10) and *rs7574865* (G/T, OR = 1.97, CI = 1.21–3.21; T/T, OR = 3.33, CI = 1.01–11.02) were associated with RA in the Polish population. *Rs4810485* was also associated with RA, however after Bonferroni’s correction was statistically insignificant. We also found an association between minor alleles of *rs2476601*, *rs2240340,* and *rs7574865* and RA (OR = 2.32, CI = 1.47–3.66; OR = 2.335, CI = 1.64–3.31; OR = 1.88, CI = 1.27–2.79, respectively). Multilocus analysis revealed an association between CGGGT and rare (below 0.02 frequency) haplotypes (OR = 12.28, CI = 2.65–56.91; OR = 3.23, CI = 1.63–6.39). In the Polish population, polymorphisms of the *PADI4*, *PTPN22,* and *STAT4* genes have been detected, which are also known risk factors for RA in various other populations.

## 1. Introduction

Rheumatoid arthritis (RA) is a chronic inflammatory disorder with a multifactorial basis; however, the etiology of RA remains unknown. It affects approximately 1% of the population and is manifested by the destruction of synovial joints, joint swelling, bone erosion, and joint tenderness [1]. It can lead to severe disability or even premature mortality [2]. The prevalence of RA varies between population [3], most likely due to population variations arising from diverse genetic backgrounds as genetic factors play an essential role and are likely 70% responsible for susceptibility to RA and expression of symptoms [3]. The increased risk of the occurrence of RA has been associated mainly with *HLA-DRB1* alleles, which are described in most genetic studies as the predominant cause of RA and are responsible for at least 30% of the total genetic basis of RA [4,5]. However, it has been suggested that single nucleotide polymorphisms (SNPs) in non-*HLA* genes can also be involved in RA pathogenesis. Large-scale genome-wide association studies (GWAS) identified risk factors for RA and described to be risk loci for also other autoimmune diseases several SNPs in genes including: protein tyrosine phosphatase non-receptor type 22 (*PTPN22*, *rs2476601*), peptidylarginine deiminase type 4 (*PADI4*, *rs2240340*), tumor necrosis factor receptor-associated factor 1 (*TRAF1*, *rs3761847*), signal transducer and activator of transcription 4 (*STAT4*, *rs7574865*), and cluster of differentiation 40 (*CD40*, *rs4810485*) [4,6]. The products of these genes are involved in the pathogenesis of RA; therefore, the role of all SNPs in RA has a rational explanation. In RA patients the serum level of TRAF1 is described as elevated and correlated with disease activity or the presence of RF antibodies [7]. *PADI4* is a gene encoding PADI4 protein, which is involved in the process of citrullination, leading possibly to the formation of autoantigens in RA patients. Anti-citrullinated protein antibodies (ACPA) are directed against posttranslational modified, citrullinated proteins. The presence of ACPA precedes the clinical onset of RA, and the level of ACPA correlates with the patient’s prognosis [8]. CD40 is a transmembrane protein expressed on antigen-presenting cells, which binds to CD40 ligand expressed on lymphocytes T. Expression of *CD40* is crucial for a threshold of activation of lymphocytes T and further steps of the immune response leading to autoantibody production and activation of macrophages [9,10]. The pathogenesis of RA can be linked to the *STAT4* gene through participation in Th17 and Th1 lineages, which are known as crucial effectors in chronic inflammatory disorders [11,12,13]. PTPN22 is known to be a master regulator of the immune system, it mediates relevant immune responses and plays a role as a negative regulator of signaling pathways mediated through the T cell receptor (TCR) and B cell receptor (BCR) [14,15].

Following these findings, an analysis of the prevalence of the mentioned polymorphisms in the Polish population is justified, since these SNPs have been evaluated in other populations and the quantity of data from the Central European population is rather limited. Therefore, assuming ethnic genetic variation, the contribution of genetic factors to the development of RA may not be identical between populations [16,17,18,19,20,21,22,23,24]. In this study, we are the first to analyze the prevalence of 5 SNPs associated with RA in the Polish population with one exception. *PADI4*.*rs2240340* was assessed in previous research [25], however, this study has a number of flaws, the most important of which is the abnormal disproportion between controls and patients (25 vs. 122) and the resulting very low power.

## 2. Results

### 2.1. Characteristics of the Study Population

We analyzed a cohort of 181 RA patients and 153 healthy controls (HC) for RA association, (clinical characteristic is presented in Table 1). The mean time of disease duration was 19 ± 10 years (from 1 to 39 years). A total of 132 patients were currently (for at least one month before blood collection) treated with methotrexate, 6 patients with sulfasalazine, and 37 patients did not receive disease-modifying anti-rheumatic drugs (DMARDs) within the last month. A total of 82 patients were taking glucocorticosteroids within the last week. In 128 patients, rheumatoid factor was positive, and anti-citrullinated protein antibodies were increased in 108 patients. In 43 patients, neither RF nor aCCP were increased. The disease activity was also assessed based on Disease Activity Score 28-joint count C reactive protein (DAS28)—CRP score (DAS < 1.7 was defined as remission, DAS > 1.7 and <2.6 was defined as low disease activity and DAS28 above 5.1 as high disease activity). All control patients had ESR and CRP within normal limits and did not have any chronic disease of inflammatory background. The genotype of 31 patients and 3 healthy subjects were unsuccessful, so the final number of subjects was 150 in both groups.

### 2.2. The Hardy–Weinberg Equilibrium Analysis

Testing for the Hardy–Weinberg (HW) principle is shown in Table 2. The genotypic distributions of all analyzed SNPs except *PADI4*.*rs2240340* were in accordance with Hardy–Weinberg equilibrium (*p* > 0.05).

### 2.3. Analysis of a Relationship between the Occurrence of RA and the Studied Polymorphic Variants of PTPN22, PADI4, TRAF1, STAT4, and CD40 Genes

Association studies were used for the analysis. These are population-based studies that determine, in part, whether a particular allele of a gene is more common in RA patients than in healthy individuals. For each polymorphism, the prevalence of each genotype compared to the general population is presented in Table 3.

We have taken into account only the most general, codominant genetic model as the mode of inheritance in RA is rather unknown and varies from autosomal dominant to recessive. The distribution of the *PTPN22*.*rs2476601*, *PADI4*.*rs2240340*, *TRAF1*.*rs3761847*, *STAT4*.*rs7574865*, *CD40*.*rs4810485* genotypes in the HC and RA groups are listed in Table 4. We observed a relationship between the occurrence of RA and almost all analyzed SNPs except *TRAF1*.*rs3761847*. *PTPN22*.*rs2476601*, *PADI4*.*rs2240340,* and *STAT4*.*rs7574865* increased the RA risk about two times. *CD40*.*rs4810485* decreased the RA risk, however, the association was not significant after Bonferroni correction.

No significant association was observed between the *TRAF1*.*rs3761847* and the incidence of RA.

### 2.4. Haplotype Analysis

Haplotypic analysis indicated a low degree of linkage disequilibrium (LD) of studied polymorphisms (Table 5).

Of the 29 possible haplotypes, 17 were present at a frequency below 0.02 and were therefore classified as rare haplotypes (Table 6). The frequencies of the other haplotypes ranged from 0.0203 to 0.24.

In multilocus analysis, the frequency of four haplotypes (TGGGG; CGGGT; TTGGG, and rare) was significantly higher in RA patients in comparison to controls (Table 7). The association remained significant even after Bonferroni correction (*p* = 0.0017) for the two haplotypes (CGGGT and rare).

### 2.5. Sample Power

The power of the sample size was estimated using a QUANTO (https://bio.tools/QUANTO, accessed on 1 May 2009) [26]. When the conditions of the sample power (α = 0.05, risk = two-fold, the number of cases = 150) were employed, we had 0.917 for *rs2240340*, 0.8855 for *rs7574865*, 0.8793 for *rs2476601*, 0.8906 for *rs3761847* and 0.9999 for *rs4810485*. Therefore, our case–control study was sufficiently powerful to study a positive correlation.

## 3. Discussion

In this study, we evaluated five SNPs occurring worldwide in different populations in genes that were previously associated with RA. We found an association between *PADI4*.*rs2240340*, *PTPN22*.*rs2476601* and *STAT4*.*rs7574865* SNPs and RA.

*PADI4*.*rs2240340* is located in the intronic region of the gene and its variants could affect the stability of the *PADI4* transcript or may alter transcriptional regulation which may shift it into its pathological activity [27]. These findings confirm those of earlier studies, such as the association with RA occurrence in different European and other populations. In 2003, Suzuki et al. for the first time described the association between *PADI4* gene polymorphisms and RA susceptibility in the Japanese population [27]. Next, *PADI4* was associated with RA occurrence in the German population [28]. A result of the meta-analysis provided by Burr et al. reported that *PADI4*.*rs2240340* was stronger associated with RA in the Asian population rather than in the European population [17]. However, there are discrepancies not only between but also within one ethnic group. For example, in the Chinese Han population, there were no significant differences between healthy control and RA [29].

*STAT4* belongs to the STATs family which are latent cytoplasmic transcription factors [30]. Studies on *STAT4* expression showed that compared to the *rs7574865* G allele, the presence of the T allele can significantly promote *STAT4* mRNA transcription and protein expression [31]. Our results are in line with others [22], wherein the Slovak population the association between *STAT4*.*rs7574865* and RA was found. The meta-analysis provided by Gao et al. based on 37 articles revealed, that when we evaluate results in terms of ethnicity *STAT4*.*rs7574865* was associated with the RA risk in Caucasians, Asians, Africans, and Mexicans [32]. Another, current meta-analysis performed by Ebrahimiyan et al. describes that the TT genotype was presented mainly in RA patients. The dominant and recessive genetic models of TT + GT vs. GG and TT vs. GT + GG both were associated with higher RA risk, respectively [33].

*PTPN22* gene encodes a protein which is known as lymphoid tyrosine phosphatase (LYP) also called protein tyrosine phosphatase non-receptor type 22 [34]. *PTPN22*.*rs2476601* (also known as *C1858T*, *R620*) leads to the alteration of amino acid residue 620 from arginine to tryptophan in the mature protein. However, its influence on the function of LYP described as a possible loss of negative regulation of T cell signaling protein remains debatable and both gain and loss of function have been suggested [14,34,35,36]. The first association with RA was described by Begovich minor T allele and RA [14]. The meta-analysis showed that there are statistically significant results in all genetic models: dominant model, recessive model, allelic model, TT vs. CC model, and CT vs. CC in Caucasians and Africans [37]. In Asian studies on the Chinese Han population by Tang et al. *rs2476601* was lacking in polymorphism with a ≤ 0.1% frequency in RA patients and healthy controls and it is suggested that is extremely rare in Asians in the context of RA studies [38]. *PTPN22* is described as one of the strongest non-*HLA* genetic predisposition factors in the RA [39] so obtaining the highest OR for this polymorphism in our Polish cohort was not surprising.

We also found an association between *rs4810485* located within the *CD40* gene and RA, however after Bonferroni’s correction it was statistically insignificant. *rs4810485*.*CD40* is located in the second intron of the *CD40* gene. It is suggested that this SNP can affect *CD40* mRNA and protein expression in B cells and monocytes, however, these results are not consistent [40,41]. *rs4810485*.*CD40* GG genotype is associated with the destruction of the joints in the ACPA-positive RA [42]. Protective effect against the development of RA has a common variant (the minor T allele) at the *CD40* and it was also observed in European populations based on meta-analysis [43]. However, other meta-analyses show that the T allele was found to be associated with RA risk in Europeans (OR 0.879 95% CI 0.848–0.901, *p* = 3 × 10^−9^) but in Asian studies show no correlation including recessive, dominant, and additive models [44]. In our results, there was no correlation in the Polish population observed under any genetic model between *rs4810485*.*CD40* and RA occurrence. Similar results were assessed in the Western Mexico population [41]. We cannot exclude that study on a larger group would pass Bonferroni’s correction.

## 4. Materials and Methods

### 4.1. Patients and Control Group

The study group included 181 patients with RA (145 women and 36 men; mean age 61 ± 13) selected from patients of the Department of Rheumatology, Medical University of Lodz, and outpatient clinic. A control group of 153 volunteers (117 women and 36 men; mean age 45 ± 16) was recruited from healthy people. The exclusion criteria only for control group were no symptoms of chronic inflammatory conditions and no RA history and the exclusion criteria for both the study and control groups were past or present malignancy. This cohort study was approved by the Institutional Bioethics Committee of the Medical University of Lodz (Lodz, Poland) (no. RNN/07/18/KE, approved date: 16 January 2018). All patients fulfilled the EULAR/ACR 2010 diagnostic criteria of RA.

### 4.2. Genotyping

Genomic DNA (gDNA) was isolated by using GeneMatrix Blood DNA Purification Kit (EURx, Gdansk, Poland) according to the manufacturer protocol. To determine the purity and concentration of gDNA we used TAKE3 plate and multi-plate reader (BioTek, Hong Kong, China) for photometric measurement. Genotypes were determined by Taqman SNP Genotyping Assay (Applied Biosystems, Foster City, CA, USA) and HOT FIREPol^®^ Probe qPCR Mix (Solis, Tartu, Estonia). Analysis was made in Bio-Rad CFX96 system (BioRad, Hercules, CA, USA) according to the manufacturer protocol. The analyzed SNPs are presented in Table 8.

### 4.3. Data Analysis

A statistical analysis of genotype frequencies, Hardy–Weinberg equilibrium, linkage disequilibrium (LD) analysis between the five SNPs, and haplotype analysis was assessed using SNPStats (https://www.snpstats.net/start.htm, accessed on 20 February 2018) [45]. Results with *p* < 0.05 were considered statistically significant. Bonferroni’s correction was applied to reduce type I error in multiple testing by multiplying the *p* values by the number of SNPs or haplotypes (five SNP—significant value was set at 0.05/5 = 0.01 for genotype analyses; 29 haplotypes—significant value was set at 0.05/29 = 0.0017 for genotype analyses). The statistical analysis determined the risk of an event (odds ratio—OR) and the confidence interval (95% CI) with the use of a linear regression model. Power calculations were conducted using Quanto 1.2.4 software [46].

## 5. Conclusions

Our study confirmed that polymorphisms involving the *PADI4* gene, whose protein is the main factor of citrullination, also occurred in the Polish RA population. Similarly, the *STAT4* gene, presented in macrophages involved in the synovitis of the joints. Moreover, in *PTPN22* loss of negative regulation of T cell signaling protein has been reported.

Our study has some limitations. First of all, our study had pilot character and further research, performed on a larger group, is needed to establish more in-depth an association between RA and studied SNPs. We cannot make an analysis on RA subgroups related to different RA features due to no desirable statistical power (>0.8). Moreover, the average age of the patient group is about 61 years, while it is 45 years for the control group. Usually, the control group and the patient should be matched in terms of age, in addition to being matched in terms of gender. However, the mean age at the time of onset of the RA is about 45 y [47] and this value is similar to our control mean age. We have an older patients group because it contains patients treated for RA for many years at our clinic but not many newly diagnosed, “fresh” RA.

In the future, to confirm our results, replication studies of a large number of cases and/or additional studies of other SNPs within five genes would be required. Moreover, it would be excellent to understand the role of these SNPs in the clinical characteristics of RA in the Polish population.

Nevertheless, our study provides possible biomarkers for RA diagnosis and provides clues for further mechanism study, especially in the Polish population.

## Figures and Tables

**Table 1:** Data present the clinical characteristics of the studied group with the mean values of parameters (±SD).

Parameters	RA Patients *n* = 181	Control Group *n* = 153
Sex	F-145 M-36	F-117 M-36
Age	61 ± 13 years	45 ± 16
Disease duration	19 ± 10 years	
Remission	yes 14; no 167	
CRP	16.3 ± 17.1	
ESR	23.9 ± 22.5	
RF	158.2 ± 211.6	
ACPA	589.1 ± 303.1	
Treatment	Methotrexate 132/181 Sulfasalazine 11/181 Leflunomide 13/50 Chloroquine 3/50 No DMARDs 37/50 Glucocorticosteroids 82/181	

CRP (C-reactive protein), ESR (erythrocyte sedimentation rate), RF (rheumatoid factor), ACPA (anti-citrullinated peptide antibodies), F-Female, M-Male.

**Table 2:** The Hardy–Weinberg equilibrium analysis.

Gene Polymorphism	*p*-Value		
	All Subjects	Control Group	Study Group
*PTPN22*.*rs2476601*	0.67	1	0.81
*PADI4*.*rs2240340*	*p* < 0.05	*p* < 0.05	1
*TRAF1*.*rs3761847*	0.34	0.61	0.41
*STAT4*.*rs7574865*	1	1	0.69
*CD40*.*rs4810485*	0.86	0.66	0.25

**Table 3:** Basic information and allele frequencies of the 5 selected SNPs in *PTPN22*, *PADI4*, *TRAF1*, *STAT4,* and *CD40* genes.

Gene SNP	Chr *	Positions **	Allele	Minor Allele Frequency	
				Case	Control
*PTPN22*.*rs2476601*	1	113834946	A/G	0.19	0.11
*PADI4*.*rs2240340*	1	17336144	C/T	0.44	0.27
*TRAF1*.*rs3761847*	9	120927961	A/G	0.45	0.41
*STAT4*.*rs7574865*	2	191099907	G/T	0.28	0.16
*CD40*.*rs4810485*	20	46119308	G/T	0.18	0.24

* Chromosome; ** Chromosome position according to the Genome Reference Consortium Human Build 38.

**Table 4:** Association of RA with the frequency of genotypes of the *PTPN22*, *PADI4*, *TRAF1*, *STAT4,* and *CD40* genes.

Gene Polymorphism	Genotype	Control Group	RA Group	OR (95% CI)	*p*-Value
*PTPN22*.*rs2476601*	G/G	119 (79%)	92 (61%)	1.00	*p* < 0.00001
	G/A	30 (20%)	50 (33%)	**2.16 (1.27–3.66)**	
	A/A	1 (1%)	8 (5%)	**10.35 (1.27–84.21)**	
	G	268 (89%)	235 (78%)	**0.43 (0.27–0.68)**	*p* = 0.0003
	A	32 (11%)	65 (22%)	**2.32 (1.47–3.66)**	
*PADI4*.*rs2240340*	C/C	96 (64%)	48 (32%)	1.00	*p* < 0.0001
	C/T	34 (23%)	74 (49%)	**4.35 (2.55–7.42)**	
	T/T	20 (13%)	28 (19%)	**2.80 (1.43–4.10)**	
	C	226 (75%)	170 (57%)	**0.42 (0.3–0.6)**	*p* < 0.0001
	T	74 (25%)	130 (43%)	**2.335 (1.64–3.31)**	
*TRAF1*.*rs3761847*	A/A	54 (36%)	42 (28%)	1.00	0.26
	A/G	75 (50%)	80 (53%)	1.37 (0.82–2.29)	
	G/G	21 (14%)	28 (19%)	1.71 (0.86–3.43)	
	A	183 (61%)	164 (55%)	0.77 (0.56–1.07)	*p* = 0.1165
	G	117 (39%)	136 (45%)	1.29 (0.93–1.79)	
*STAT4*.*rs7574865*	G/G	104 (69%)	78 (52%)	1.00	0.0059
	G/T	42 (28%)	62 (41%)	**1.97 (1.21–3.21)**	
	T/T	4 (3%)	10 (7%)	**3.33 (1.01–11.02)**	
	G	250 (83%)	218 (73%)	**0.53 (0.36–0.79)**	*p* = 0.0018
	T	50 (17%)	82 (17%)	**1.88 (1.27–2.79)**	
*CD40*.*rs4810485*	G/G	86 (6%)	100 (1%)	1.00	0.027 *
	G/T	54 (37%)	48 (34%)	0.76 (0.47–1.24)	
	T/T	10 (57%)	2 (65%)	0.17 (0.04–0.81) *	
	G	226 (75%)	248 (83%)	1.56 (1.05–2.33) *	*p* = 0.02 *
	T	74 (25%)	52 (17%)	0.64 (0.43–0.95) *	

* Not statistically important after applying Bonferroni’s correction.

**Table 5:** Linkage disequilibrium analysis of the 5 selected SNPs in *PTPN22*, *PADI4*, *TRAF1*, *STAT4,* and *CD40* genes. D’ statistic is presented. *** are placed instead of repeated genes or values.

Gene Polymorphism	*PADI4* *rs2240340*	*STAT4* *rs7574865*	*PTPN22* *rs2476601*	*TRAF1* *rs3761847*	*CD40* *rs4810485*
*PADI4* *rs2240340*	***	0.1872	0.1236	0.1447	0.0031
*STAT4* *rs7574865*	***	***	0.1561	0.0894	0.0494
*PTPN22* *rs2476601*	***	***	***	0.1296	0.0485
*TRAF1* *rs3761847*	***	***	***	***	0.1091
*CD40* *rs4810485*	***	***	***	***	***

**Table 6:** Haplotype frequencies of the 5 selected SNPs in *PTPN22*, *PADI4*, *TRAF1*, *STAT4,* and *CD40* genes estimation. * described haplotypes present at frequency below 0.02.

Number	*PADI4* *rs2240340*	*STAT4* *rs7574865*	*PTPN22* *rs2476601*	*TRAF1* *rs3761847*	*CD40* *rs4810485*	Total
1	C	G	G	A	G	0.2405
2	C	G	G	G	G	0.1312
3	T	G	G	G	G	0.0816
4	T	G	G	A	G	0.0808
5	C	G	G	A	T	0.0596
6	C	G	G	G	T	0.0448
7	C	T	G	A	G	0.0421
8	T	T	G	A	G	0.0407
9	C	G	A	A	G	0.0359
10	C	T	G	G	G	0.0275
11	T	T	G	G	G	0.0223
12	T	G	G	A	T	0.0203
Rare	*	*	*	*	*	0.1728

**Table 7:** Association of RA with the frequency of haplotype of the 5 selected SNPs in *PTPN22*, *PADI4*, *TRAF1*, *STAT4,* and *CD40*. * described haplotypes present at frequency below 0.02.

Number	*PADI4* *rs2240340*	*STAT4* *rs7574865*	*PTPN22* *rs2476601*	*TRAF1* *rs3761847*	*CD40* *rs4810485*	OR (95% CI)	*p*-Value
1	C	G	G	A	G	1	---
2	C	G	G	G	G	0.53 (0.24–1.18)	0.12
3	T	G	G	G	G	3.27 (1.23–8.72) ^#^	0.019 ^#^
4	T	G	G	A	G	0.47 (0.18–1.26)	0.13
5	C	G	G	A	T	0.42 (0.13–1.36)	0.15
6	C	G	G	G	T	**12.28 (2.65–56.91)**	0.0015
7	C	T	G	A	G	0.28 (0.07–1.19)	0.086
8	T	T	G	A	G	1.07 (0.29–3.92)	0.92
9	C	G	A	A	G	0.33 (0.06–1.72)	0.19
10	C	T	G	G	G	0.16 (0.02–1.18)	0.072
11	T	T	G	G	G	0.05 (0.00–0.61) ^#^	0.02 ^#^
12	T	G	G	A	T	0.38 (0.02–6.87)	0.51
Rare	*	*	*	*	*	**3.23 (1.63–6.39)**	0.0009

^#^ Not statistically important after applying Bonferroni’s correction

**Table 8:** Single nucleotide polymorphisms analyzed in this study.

Gene	RS Number ^1^	Assay ID ^2^
*PTPN22*	*rs2476601*	C__16021387_20
*PADI4*	*rs2240340*	C__16176717_10
*TRAF1*	*rs3761847*	C___2783640_10
*STAT4*	*rs7574865*	C__29882391_10
*CD40*	*rs4810485*	C___1260190_10

^1^ According to dbSNP database; ^2^ related to Applied Biosystems™.

## Data Availability

The data presented in this study are available on request from the corresponding author. The data are not publicly available due to law regulations in country of corresponding author.
